# Annual Foot Exams and Incident Amputation

**DOI:** 10.1093/geroni/igaa057.723

**Published:** 2020-12-16

**Authors:** Latricia Allen, Anjali Khakharia, Lawrence Phillips, Theodore Johnson, Constance Uphold, Molly Perkins, Camille Vaughan

**Affiliations:** 1 Emory University School of Medicine, Decatur, Georgia, United States; 2 Emory University, Decatur, United States; 3 Emory University School of Medicine, Atlanta, Georgia, United States; 4 eVA Geriatric Research Education and Clinical Center, Decatur, Florida, United States; 5 Emory University, Atlanta, Georgia, United States

## Abstract

Diabetes-related lower extremity amputations (LEA) are high cost and high prevalence. Individuals with complications such as neuropathy, foot deformity, history of diabetic foot ulcer or LEA increased morbidity and mortality.1 Current national recommend a foot exam for individuals with diabetes annually or more often depending on risk for LEA.2, The purpose of this pilot study was to examine the relationship between annual foot exams and incident lower extremity amputation in a large Veteran cohort. We conducted a secondary analysis of a national VA Diabetes administrative dataset registry for Veterans with diabetes aged 65 and older during the period of fiscal year 2002-2014 (n=1,544,654; mean age 77.6 years; 97.9 % male). Using logistic regression, we examined the association between annual foot exams and incident LEA. Our analysis was adjusted for demographics, comorbidities, and LEA foot risk. The study included 18,759 (1.21%) Veterans with incident LEA and foot exams, 2,234 (0.14%) Veterans with incident LEA without foot exams. Median age range was 65-75 years old. Gangrene, osteomyelitis, foot ulcers, and neuropathy were the covariates with the highest risk of incident LEA with foot exam. Foot exams did not reduce the risk of LEA when examining Veterans with incident LEA (unadjusted OR of 1.62 (CI 1.56 - 1.69), p<.0001 and adjusted OR was 1.77 (CI 1.69 -1.86), p<.0001. Annual foot exams were not protective for LEA in Veterans with foot exams and incident LEA. Additional research is warranted to examine this relationship considering the effect of early intervention on LEA risk.

